# Prophylactic Zinc Administration Combined with Swimming Exercise Prevents Cognitive-Emotional Disturbances and Tissue Injury following a Transient Hypoxic-Ischemic Insult in the Rat

**DOI:** 10.1155/2022/5388944

**Published:** 2022-05-20

**Authors:** Ana-Karina Aguilar-Peralta, Alejandro Gonzalez-Vazquez, Constantino Tomas-Sanchez, Victor-Manuel Blanco-Alvarez, Daniel Martinez-Fong, Juan-Antonio Gonzalez-Barrios, Ilhuicamina Daniel Limon, Lourdes Millán-Perez Peña, Gonzalo Flores, Guadalupe Soto-Rodriguez, Eduardo Brambila, Jorge Cebada, Viridiana Vargas-Castro, Bertha Alicia Leon-Chavez

**Affiliations:** ^1^Facultad de Ciencias Químicas, Benemérita, Universidad Autónoma de Puebla, 14 Sur y Av. San Claudio, 72570 Puebla Pue, Mexico; ^2^Facultad de Enfermería, Benemérita, Universidad Autónoma de Puebla, 27 Sur 1304, Col. Volcanes, 72410 Puebla Pue, Mexico; ^3^Departamento de Fisiología, Biofísica y Neurociencias, Centro de Investigación y de Estudios Avanzados del Instituto Politécnico Nacional, Apartado Postal 14-740, 07000 México D.F., Mexico; ^4^Nanoparticle Therapy Institute, 404 Avenida Monte Blanco, Aguascalientes 20120, Mexico; ^5^Laboratorio de Medicina Genómica, Hospital Regional 1° de Octubre, ISSSTE Avenida, Instituto Politécnico Nacional #1669, 07760 México D.F, Mexico; ^6^Centro de Química, ICUAP, Benemérita Universidad Autónoma de Puebla, 14 Sur y Av. San Claudio, 72570 Puebla, Mexico; ^7^Instituto de Fisiología, Benemérita Universidad Autónoma de Puebla, 14 Sur y Av. San Claudio, 72570 Puebla, Mexico; ^8^Facultad de Medicina, Benemérita Universidad Autónoma de Puebla, 13 Sur 2702, Col. Volcanes, 72410 Puebla Pue, Mexico

## Abstract

Exercise performance and zinc administration individually yield a protective effect on various neurodegenerative models, including ischemic brain injury. Therefore, this work was aimed at evaluating the combined effect of subacute prophylactic zinc administration and swimming exercise in a transient cerebral ischemia model. The prophylactic zinc administration (2.5 mg/kg of body weight) was provided every 24 h for four days before a 30 min common carotid artery occlusion (CCAO), and 24 h after reperfusion, the rats were subjected to swimming exercise in the Morris Water Maze (MWM). Learning was evaluated daily for five days, and memory on day 12 postreperfusion; anxiety or depression-like behavior was measured by the elevated plus maze and the motor activity by open-field test. Nitrites, lipid peroxidation, and the activity of superoxide dismutase (SOD) and catalase (CAT) were assessed in the temporoparietal cortex and hippocampus. The three nitric oxide (NO) synthase isoforms, chemokines, and their receptor levels were measured by ELISA. Nissl staining evaluated hippocampus cytoarchitecture and Iba-1 immunohistochemistry activated the microglia. Swimming exercise alone could not prevent ischemic damage but, combined with prophylactic zinc administration, reversed the cognitive deficit, decreased NOS and chemokine levels, prevented tissue damage, and increased Iba-1 (+) cell number. These results suggest that the subacute prophylactic zinc administration combined with swimming exercise, but not the individual treatment, prevents the ischemic damage on day 12 postreperfusion in the transient ischemia model.

## 1. Introduction

Cerebrovascular disease (CVD) is caused by a temporary or sustained deprivation of oxygen and nutrients in one or more brain areas, leading to cerebral parenchyma injury [1]. This disease affects 15 million people worldwide every year and is one of the leading causes of permanent disability and mortality [2]. In addition, ischemia is a more frequent cause than the hemorrhagic process in CVD [3]. The ischemic process disrupts the blood flow, alters cell homeostasis, and induces immediate cell death by necrosis in the infarct core [4]. Furthermore, in the penumbra zone, the ischemic process triggers deleterious events such as excitotoxicity (release of glutamate and calcium), nitrosative and oxidative stress [5], lytic enzyme activation (proteases, lipases, and nucleases), dynamic cytoskeleton alteration [6], and neuroinflammation [7].

Severe cerebral ischemic damage has been reproduced in rodents with cerebral artery occlusion (MCAO) or with common carotid artery occlusion (CCAO) for more than one hour (up to 120 min) [8, 9], which promotes the formation of extensive infarct zones in the brain [10, 11]. Models with shorter occlusion times, such as CCAO for 10 min, do not promote an infarct core [12, 13] but cause learning and memory loss at 12 days postreperfusion [12, 14, 15]. In the 30 min CCAO model, although some authors have reported that it does not cause infarct volume at 24 h postreperfusion [7, 16], others have proven that it mimics the physiopathology of human ischemic-type CVD [17]. The CCAO for 30 min can cause an infarct core and the penumbra zone, affecting several brain regions such as the lateral neocortex, striatum, and ipsilateral hippocampus [18, 19]. In the early phase of 30 min CCAO, it has been reported that the alteration of energy metabolism [20, 21] detonates oxidative/nitrosative stress [16] and neuroinflammation [22, 23]. The humoral response is characterized by the expression of proinflammatory cytokines (IL-1*β*, TNF-*α*, and IFN-*γ*) [22], adhesion molecules (ICAM-1 and VCAM-1) [21], and chemokines (CCL2, CCL3, CXCL1, CXCL13, and CX3CL1) whose levels maintain up to 5 days postreperfusion [24]. The cellular response includes an increase in microglia number [25, 26] and the presence of infiltrated macrophages, neutrophils, and B lymphocytes [22] in the infarct core [27–29]. Those cellular events correlate with neuronal cell death, and the maximum increase occurs on day 7 postreperfusion [30]. Posteriorly, regeneration processes originated in the subgranular zone (SGZ) [26], and removal of the infarct by astrocytes and microglia occurs until day 14 postreperfusion [27]. However, the neuropsychological alterations prevail [27–29] from 21 days [30] to 90 days postreperfusion [31].

Nevertheless, the constant release of proinflammatory cytokines, chemokines, and adhesion molecules can elicit apoptosis and affect neuroplasticity, which causes cognitive deficits such as learning and memory loss and emotional disturbances such as anxiety or depression-like behavior [32]. Interestingly, some reports have shown that cognitive activity or motor performance protects against neurodegenerative diseases, improving cerebral functionality [33, 34]. Swimming exercise, for example, has shown neuroprotection in animal models of Alzheimer [35], spinal muscular atrophy [36], depression [37], and amyotrophic lateral sclerosis, thorough increasing the production and release of growth factors [38]. However, the beneficial effect of exercise performance depends on factors like intensity and frequency to trigger efficient cellular adaptation mechanisms to oxidative stress and increase the antioxidative response stimulated by mitochondrial biogenesis [39, 40].

An increasing number of reports support the neuroprotector effect of zinc administration prophylactically [11, 41, 42] or postreperfusion [43]. However, zinc administration can also exacerbate ischemia-induced cerebral injury depending on the dose and time of administration. The neuroprotective effect was found in subacute prophylactic ZnCl_2_ administration (2.5 mg/kg/24 h for 4 days, i.p., before 10 min CCAO) [11, 41] and hyperacute ZnCl_2_ administration at a dose of 20 mg/kg, i.p., 30 min before 3 min CCAO [44]; 10.9 mg/kg equivalent of zinc, i.p. 30 min before 2 h ischemia [45]; and 6 mg/kg, i.v., after 60 min of 2 h medial carotid artery occlusion (MCAO) [46]. On the contrary, chronic prophylactic ZnCl_2_ administration at a dose of 0.5 mg/kg/15 days, i.p. [47], or zinc protoporphyrin IX treatment can exacerbate ischemia-induced cerebral injury [48–50]. Other factors that influence zinc neuroprotection are the time (3 min to 2 h) postreperfusion and the modality of obliteration (unilateral or bilateral) [44, 45] or injury size in the case of traumatic brain injury (TBI) models [51, 52].

The main beneficial effect of zinc is to promote antioxidant and anti-inflammatory actions against ischemic damage. It modulates the formation of ROS by inhibiting NMDAR through its binding to the GluN2A subunit [53, 54]; decreases the reactivity of O_2_^−^ due to a structural part of the cytosolic superoxide dismutase (SOD1) [55]; regulates the transcription of reduced glutathione (GSH), SOD, reduced glutathione transferase (GST), and heme oxygenase- (HO-) 1 through Nrf2 [56]; induces BDNF expression [57]; converts pro-BDNF to mature BDNF by activation of zinc-dependent MMPs [58]; prevents the formation of the complex IKK through the interaction of Zn^2+^ via influx by Zip8 [59], or the A20 protein [60], inhibiting the NF-*κ*B signaling pathway [61] and decreasing neuroinflammation; and increases the density of neuronal precursor cells after a TBI [62], promoting neurogenesis [63, 64]. Furthermore, zinc through microglia-expressed ZEB1 can regulate neuroinflammation after stroke, decreasing neutrophil chemoattraction by CXCL1 [65].

It has been reported that subacute prophylactic zinc administration in the 10 min CCAO model has a neuroprotective effect, reducing nitrosative stress and cell death [11], increasing chemokine and growth factor levels, and improving learning and memory in the Morris Water Maze (MWM) test [41]. On the contrary, zinc deficiency caused anxiety-like behavior in rats submitted to MWM [66]. Besides, chronic prophylactic zinc (0.2 mg/kg by 14 days) combined with therapeutic selenium administration has a neuroprotective and antioxidant effect on hypoxic-ischemic brain events postreperfusion [42].

Based on the neuroprotection of exercise performance and subacute prophylactic zinc administration, this work was aimed at evaluating whether the combined neuroprotection of prophylactic zinc administration (2.5 mg/kg/24 h for four days) and swimming exercise through MWM can be effective in brain injury caused by 30 min CCAO. The biochemical parameters studied were NO and malondialdehyde and 4-hydroxy-alkenal (MDA+4-HDA) levels and chemokine (CCL2, CXCL1, CXCL13, and CX3CL1) and nitric oxide synthase (NOS-1, NOS-2, and NOS-3) protein levels, measured by ELISA. In addition, Nissl staining and Iba-1 immunohistochemistry were performed on day 12 postreperfusion. At this time, cognition was also evaluated, whereas emotional disturbances were evaluated on day 30 postreperfusion.

## 2. Methodology

### 2.1. Experimental Animals

Male Wistar rats (bodyweight 180-240 g) were obtained from the Animal Production Unit of CINVESTAV and maintained in suitable rooms with controlled conditions of temperature (22 ± 3°C) and light-dark cycles (12 h–12 h; light onset at 07:00). The animals received food (Laboratory Autoclavable Rodent Diet 5010, 130 ppm of zinc) and drinking water *ad libitum*. All procedures were carried out according to the current Mexican legislation, NOM-062-ZOO-1999 (SAGARPA), based on the Guide for the Care and Use of Laboratory Animals, NRC. The experimental procedures were approved by the Institutional Animal Care and Use Committee with protocol number 09-102. All experimental processes were made to minimize animal suffering.

### 2.2. Experimental Groups

The animals were randomly divided into the following groups: (1) healthy, without swimming exercise treatment, or surgery to normalize the effect of all treatments; (2) MWM, control of swimming exercise trained in MWM; (3) Zn, control with chloride zinc (2.5 mg/kg of body weight, equivalent to 1.2 mg atomic zinc) administration [41]; (4) CCAO, rats with 30 min left CCAO; and (5) Zn+CCAO, prophylactic zinc administration and CCAO.

### 2.3. Experimental Design

**Figure 1:**
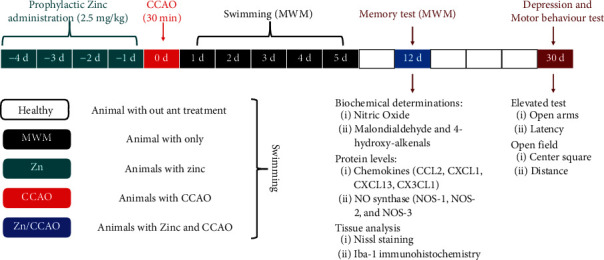


Experimental diagram of the procedures performed, zinc administration, common carotid artery obliteration (CCAO), biochemical determinations, and tests is shown to evaluate cognitive and motor abilities (Figure 1).

### 2.4. Common Carotid Artery Obliteration (CCAO)

The animals were maintained in a sterile room, and surgical instruments were sterilized. Posteriorly, animals were anesthetized with a mixture of ketamine (70 mg/kg) and xylazine (6 mg/kg) at a dose of 200 *μ*L/100 g of body weight, i.p. After an incision of 0.5 cm long midline skin in the neck area, the left common carotid artery was carefully dissected and occluded for 30 min with a clamp (Bulldog Clamps, INS6000119; Kent Scientific Corporation; Torrington, CT, USA). Upon completion of the occlusion time, the artery reperfusion was visually verified, and the incision was sutured with a 3-0 silk thread (Atramat; Ciudad de Mexico, Mexico). The animals were kept in an individual cage until their complete recovery in the surgery room and then transferred to a temporary vivarium. The animals after experimental processes were euthanized on day 12 postreperfusion using sodium pentobarbital at a dose of 60 mg/kg of body weight [41, 42].

### 2.5. Morris Water Maze (MWM)

MWM pool was used to submit the rats to physical exercise as reported previously [67, 68]. Additionally, this test was utilized to evaluate learning and memory. All experimental groups were trained at 24 h after CCAO with MWM to evaluate the learning and memory. The swimming pool was divided into four quadrants, North (N), West (W), South (S), and East (E), and two clues were placed on the inner walls of the tank, which serve as the orientation and location inside the platform. The MWM consisted of a circular tank (swimming pool) of 150 cm diameter and 80 cm height; the escape platform measuring 10 cm in diameter was placed in the quadrant southeast. The tank will be filled with water (19-22°C) one centimeter above the platform height to ensure that rats cannot see the escape platform.

The learning test consisted of four probes per day, one for each quadrant, giving 60 seconds (s) to find the platform (escape latency). Once the rats completed the trial, they remained on the platform for 30 s. Then, they were removed from the platform and waited 30 min to start the subsequent trial. The order of performance of the quadrants was N, W, S, and E, for five consecutive days.

The memory test was evaluated seven days after the last day of the learning test. The memory test was carried out for 60 s in the N quadrant, counting the number of times that the rat passed by the escape platform and the time in which it was localized [42].

### 2.6. Elevated Plus Maze

The elevated plus maze was used to assess the depressive-like behavior and anxiety of the rats. The maze was placed 90 cm from the ground and consisted of two open arms (25 × 5 × 1 cm; white color) and two closed arms (25 × 5 × 16 cm; black color) connected to a central platform (5 × 5 cm). The open arms had a wall of 1 cm to reduce the risk of falls, whereas the closed arms had a wall of 16 cm to cover the entire arm.

The test was carried out for 5 min, and the number of times that the animal entered the open and closed arms and the time to remain on them were recorded. In addition, the urination and defecation numbers were also quantified.

### 2.7. Open Field

The open-field test was also used to assess the unrestricted mobility of rats. The evaluations were carried out in a square wood box of 60 cm divided into nine quadrants per side and 70 cm high. The number of times the subject passed through the central quadrant (quadrant 5) and the distance traveled (total number of quadrants visited multiplied by 20 cm) during 5 min were registered.

### 2.8. Nitric Oxide Quantification

The NO production was assessed by the accumulation of nitrites (NO_2_^−^) by the modified technique of Griess as described previously [69]. The cerebral cortex and hippocampus were obtained 1 h after the memory test (*n* = 5 rats in each group), mechanically homogenized in phosphate-buffered saline solution pH 7.4 (PBS), and centrifuged at 12,500 rpm for 30 min at 4°C by using a Z216MK microcentrifuge (HERMLE Labortechnik; Wehingen, Germany). Briefly, the NO concentration was measured in 10 *μ*L of supernatant after the addition of 10 *μ*L of Griess reagent (composed of equal volumes of 0.1% N-(1-naphthyl) ethylenediamine dihydrochloride and 1.32% sulfanilamide in 60% acetic acid). In addition, the optical density in a 2 *μ*L sample was measured with a NanoDrop 1000 Spectrophotometer (Thermo Fisher Scientific, Bancroft Building, Wilmington, USA) at 540 nm, and the optical density values were interpolated from the standard curve of NaNO_2_ (1 to 10 *μ*M) to obtain NO concentration.

### 2.9. Lipid Peroxidation Quantification

Malondialdehyde (MDA) and 4-hydroxyalkenals (4-HDA) as an index of lipid peroxidation were measured in each group (*n* = 5), following the procedure described previously [69]. The colorimetric reaction was performed in 200 *μ*L of the supernatant supplemented with 650 *μ*L of 10.3 mM N-methyl-2phenyl-indole (Sigma-Aldrich; Saint Louis, MO, USA), which was previously diluted in a mixture of acetonitrile: methanol (3 : 1) and 150 *μ*L of methanesulfonic acid (Sigma-Aldrich; Saint Louis, MO, USA). This reaction mixture was vortexed and incubated at 45°C for 1 h and then centrifuged at 3000 rpm for 10 min. The absorbance of the reaction in supernatant was read at 586 nm m with a SmartSpec 3000 spectrophotometer (Bio-Rad; Hercules, CA, USA). The absorbance values were interpolated from a standard curve of 1,1,3,3-tetramethoxypropane (10 mM stock) in the concentration range of 0.25 to 5 *μ*M to calculate the MDA+4‐HDA level in the samples.

### 2.10. Superoxide Dismutase (SOD) Activity

Total SOD activity was quantified directly in the spectrophotometer quartz cuvette containing 8 *μ*L pyrogallol (initial substrate), 800 *μ*L of Tris-HCl buffer solution (pH 8.2), and 15 *μ*L of EDTA. After 30 s, 60 *μ*L of the supernatant was added, and the absorbance was measured immediately every minute for 3 minutes at 420 nm using a spectrophotometer (Lambda EZ-150; PerkinElmer Company; Waltham, MA, USA). The results were reported as the U min^−1^/mg protein. The equations are detailed in [69].

### 2.11. Catalase (CAT) Activity

CAT activity was quantified directly in the spectrophotometer quartz cuvette containing 50 *μ*L of the supernatant and 655 *μ*L of PBS 1X. The reaction was initiated by adding 330 *μ*L of H_2_O_2_ (30 mM), and the absorbance was measured at 240 nm every 30 s for 2 minutes with a spectrophotometer (Lambda EZ-150; PerkinElmer Company; Waltham, MA, USA). The results were reported as the U min^−1^/mg protein [69].

### 2.12. 2, 3, 5-Triphenyltetrazolium Chloride (TTC) Staining

The tetrazolium salt assays measure mitochondrial activity associated with the electron transport chain, given the rapidity with which most dehydrogenase systems reduce it. Upon reduction, tetrazolium precipitates in the form of a deep red water-insoluble complex. The experimental animals were anesthetized and perfused intracardially with 0.9% isotonic saline solution (SSI); the brains were extracted and coronally sectioned at intervals of 2 mm; then, the sections were incubated for 15 minutes at 37°C in 1% of TTC solution; posteriorly, the tissues were placed overnight in 4% paraformaldehyde solution. After staining, photographs of the brain sections were taken.

### 2.13. Nissl Staining

Brain slices of 40 *μ*m thick were placed on gelatinized slides, washed with distilled water, and stained with a 0.1% cresyl violet solution (Merck KGaA; San Luis, Missouri, USA) in a 0.25% acetic acid solution for 30 min. Subsequently, the sections were dehydrated in increasing concentrations of ethanol and rinsed with xylene (Hycel; CDMX, Mexico), and Entellan resin (Merck KGaA; Darmstadt, Germany) was used as a mounting medium [11].

### 2.14. Immunohistochemical Staining

The brains were cryoprotected with 30% sucrose and then cut into 40 *μ*m thick sagittal sections equidistant 1 : 6. Then, the slices were washed with TBS-1X (Trizma base buffer, pH 7.4) and preincubated with 3% H_2_O_2_ solution for 30 min to block endogenous peroxidase. After removing excess H_2_O_2_ with triplicated TBS-1X washes, unspecific binding sites were blocked with TBS ++ (TBS-1X, Triton X-100, and goat serum) for 30 min at room temperature (RT), and then, the slices were incubated with a primary anti-Iba-1 rabbit antibody (FUJIFILM Wako Chemicals USA Corporation 019-19741) overnight at 4°C. Later, the staining was accomplished using the ABC peroxidase system (the secondary antibody conjugated with biotin and 3,3′diaminobenzidine (DAB) as a developer). Finally, micrographs of Iba-1 (+) cells were taken with a 20x objective of a microscope (DMLS 2000, Leica Microscope) for further analysis with ImageJ software (RRID: SCR_003070, National Institute of Health).

### 2.15. ELISA Immunoassay

The ELISA plate of 96 wells (Corning, Camelback, Rd, Glendale, USA) was sensitized with five *μ*g of total protein from the homogenate of the samples and completed to a final volume of 100 *μ*L with carbonate buffer for 24 h at 4°C. Then, the ELISA plates were washed with PBS 1X containing 0.1% Tween 20, and the unspecific binding sites were blocked with 0.5% fetal bovine serum for 30 min. Subsequently, the wells were washed with PBS-0.1% Tween 20, and the primary antibody (dilution 1 : 500) was added and incubated for 24 h at 4°C. The primary antibodies were mouse anti-nNOS (Sigma Aldrich, SAB4502010), rabbit anti-iNOS (Sigma Aldrich, SAB4502012), rabbit anti-eNOS Rabbit (Sigma Aldrich SAB4502013), rabbit anti-CCL2 (Abcam ab9779), rabbit anti-CXCL1 (Abcam ab9772), rabbit anti-CXCL13 (Abcam ab112521), rabbit anti-CX3CL1 (ThermoFísher SCIENTIFIC PA1-29224), rabbit anti-CCR2 (Abcam ab21667), and rabbit anti-CXCR2 (Abcam ab14935). Subsequently, the wells were washed with PBS-0.1% Tween 20, and the secondary polyclonal antibody goat anti-rabbit and goat anti-mouse (dilution 1: 1000; Pierce Technology Co.; Holmdel, NJ, USA) was added. Then, the secondary antibodies conjugated with horseradish peroxidase were incubated for 3 h at RT (1 : 5000, Pierce Technology Co. Holmdel, NJ, USA), and finally, they were washed with 1X PBS-1% Tween 20, and the ABTS (2,2′-azino-bis- (3-ethyl benzyl-thiazoline-6-sulfonic acid) substrate was incubated for 15 min. The reaction on the plate was read in an ELISA reader (Bio-Rad Benchmark) at 415 nm.

### 2.16. Hierarchization Score of the Protective Effect of Pharmacological Strategies (HSPEPS)

The values of cognitive tests, histopathology, and biochemical probes were expressed according to the modified HSPEPS [69], indicating the efficacy of one or more pharmacological strategies (Register # MX2020010357) treatments. A value lower than healthy was zero, equal to healthy was 1, and higher than healthy was 1.5 or 2, depending on the increasing grade (Table 1). HSPEPS was constructed with the sum of all performance scores of each treatment and study (Table 2).

### 2.17. Statistical Analysis

The values are mean ± standard error of the mean (SEM). The difference of escape latency was analyzed with two-way ANOVA and Bonferroni's post hoc multiple comparisons tests, whereas the escape latency on the memory test was analyzed with Kruskal-Wallis and Dunn's post hoc. The elevated plus maze and open-field results were analyzed with Student's *t*-test and Welch's post hoc. One-way ANOVA and Dunnett's post hoc were used to analyze the biochemical results, the optical density of Nissl stain, and the number of Iba-1-positive cells in hippocampal areas. All statistical analyses were performed with the Prism software (GraphPad Prism; San Diego, CA, USA; RRID: SCR_0158070). The experimental values were compared with their respective healthy (∗), MWM group (*Φ*), and CCAO group (†). *P* < 0.05 was significant.

## 3. Results

**Figure 2:**
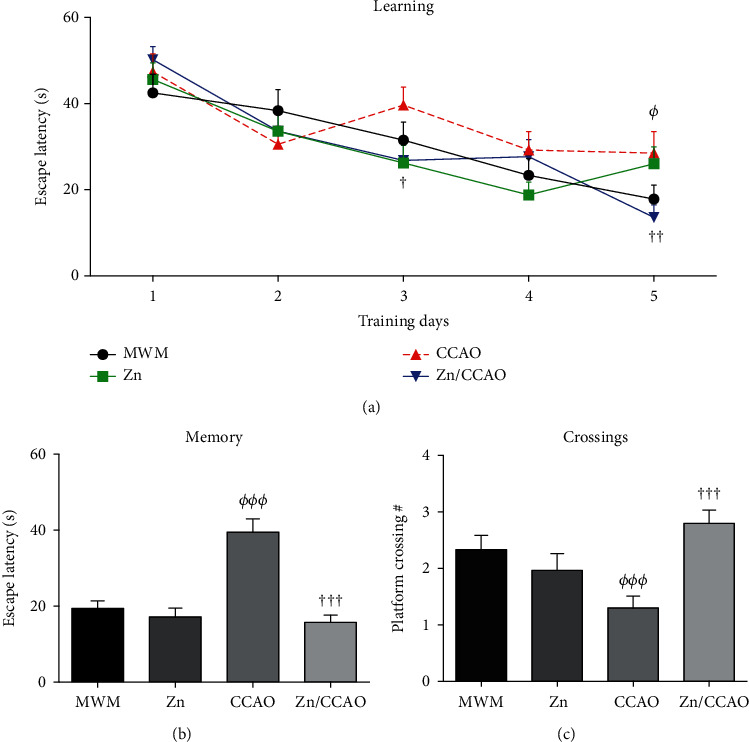
The subacute prophylactic zinc administration prevents the cognitive deficit caused by a 30 min CCAO. The values are the mean ± SEM (*n* = 21 to 30) and analyzed with two-way ANOVA and Bonferroni's post hoc multiple comparisons. In addition, Kruskal-Wallis with Dunn's post hoc test was used to analyze the escape latency in the memory test. *P* < 0.05, *^Φ^*compared with the MWM group and ^†^compared with the CCAO group.

CCAO caused a loss of acquisition of spatial learning revealed by the increased escape latency (55.4 ± 27.09%; *^Φ^P* = 0.0407) on day 5 of training compared with the MWM group (Figure 2(a)). In contrast, subacute prophylactic zinc administration significantly decreased the escape latency on days 3 (32.38 ± 9.46%, ^†^*P* = 0.0261) and 5 (52.38 ± 10.21%, ^††^*P* = 0.0099) compared with the CCAO group. The Zn group did not show a difference in spatial learning acquisition compared with the MWM group (Figure 2(a)).

Long-term memory test on day 12 postreperfusion showed that CCAO increased the escape latency by 103.44 ± 17.81% (*^ΦΦΦ^P* = 0.0001), compared with the MWM group (Figure 2(b)). On the contrary, the subacute prophylactic zinc treatment reverted (44.16 ± 8.84%, ^†††^*P* = 0.0249) the CCAO-induced increase in escape latency (Figure 2(b)). Furthermore, the CCAO group showed decreased number of platform crossings (44.16 ± 8.84%; *^Φ^P* = 0.0249) compared with the MWM group, which was prevented (114.88 ± 17.8%; ^†††^*P* = 0.0001) by the subacute prophylactic zinc treatment (Figure 2(c)). These results show that prophylactic zinc administration prevents the cognitive deficit induced by 30 min CCAO.

**Figure 3:**
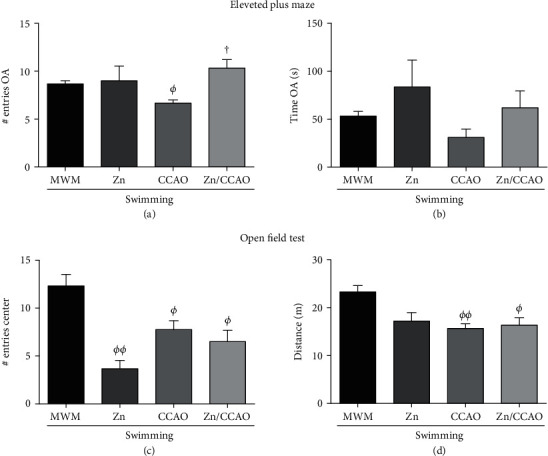
The subacute prophylactic zinc administration prevented emotional disturbance but not mobility alterations in rats with a 30 min CCAO on day 30 postreperfusion. The values are mean ± SEM (*n* = 4) and analyzed with *t*-test and Welch's post hoc. *P* < 0.05, *^Φ^*compared with the MWM group and ^†^compared with the CCAO group. OA: open arms.

The elevated plus maze (EPM) test on day 30 postreperfusion showed that CCAO decreased the number of entries to the open arms (OA) (23.07 ± 3.84%; *^Φ^P* = 0.0132) compared with the MWM group (Figure 3(a)). This effect was prevented by the subacute prophylactic zinc administration (55 ± 13.22%; ^†^*P* = 0.0399) compared with the CCAO group (Figure 3(a)). However, no statistically significant difference was found in the time of permanency in the OA of EPM (Figure 3(b)). In addition, neither group showed statistically significant differences in the number of urinations and defecations (data not shown).

The open-field test showed that CCAO also affected rat mobility. Both the distance traveled (32.83 ± 4.38%; *^ΦΦ^P* = 0.0097) and entries to the central quadrant (37.16 ± 7.67%; *^Φ^P* = 0.0379) were less than those of the MWM group (Figures 3(c) and 3(d)). Those alterations were not modified by subacute prophylactic zinc administration (Figures 3(c) and 3(d)).

The HSPEPS analysis on the cognitive tests showed that the CCAO+MWM group had a lesser value (0.5) than the MWM group (4), and the Zn+CCAO+MWM group yielded values closer to basal values (3.5) of the MWM group (Table 3).

**Figure 4:**
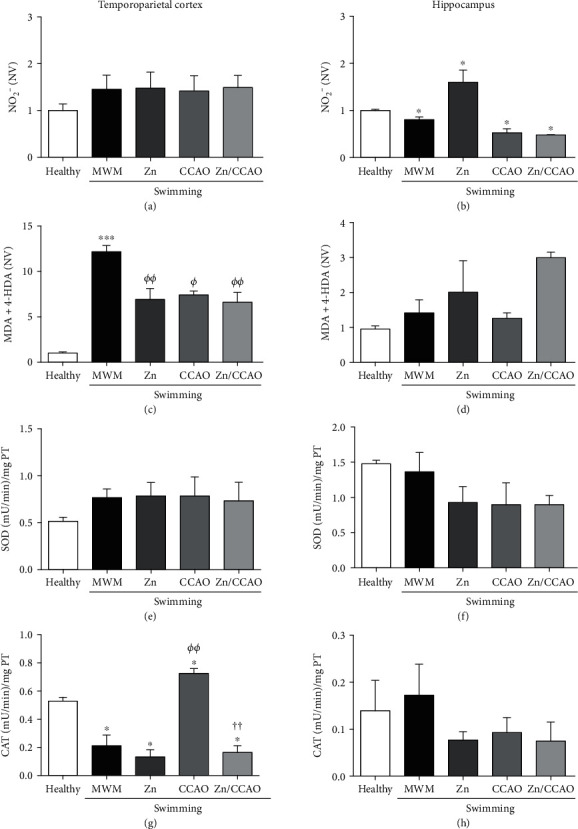
The subacute prophylactic zinc administration and swimming prevented nitrosative stress and increased catalase activity on day 12 postreperfusion only in the temporoparietal cortex of rats with 30 min CCAO. The values are mean ± SEM (*n* = 5) and analyzed with one-way ANOVA and Dunnett's post hoc multiple comparison test. *P* < 0.05, ^∗^compared with the Healthy group, *^Φ^*compared with the MWM group, and ^†^ compared with the CCAO group; NV: normalized values.

In the temporoparietal cortex, nitrite levels were not different among all experimental groups on day 12 postreperfusion (Figure 4(a)). Meanwhile, in the hippocampus, only the zinc group increased the nitrite levels, while the others groups decreased compared with the healthy group (Figure 4(b)). However, the MDA+4‐HDA levels were significantly elevated in the temporoparietal cortex of all groups compared with the healthy group. The maximum increase was found in the MWM group (1118.15 ± 67.31%, ^∗∗∗^*P* = 0.0001), whereas the increase was 591.32 ± 120.67% (^∗∗^*P* = 0.0025) in the zinc group, 643.21 ± 40.94 (^∗∗^*P* = 0.0022) in the CCAO group and 560.34 ± 108.89 (^∗∗^*P* = 0.0038) in the Zn+CCAO group (Figure 4(c)). In the hippocampus, MDA+4-HDA levels were not different among all groups (Figure 4(d)).

The SOD activity in the temporoparietal cortex (Figure 4(e)) and hippocampus (Figure 4(f)) did not show a statistical difference among all groups on day 12 postreperfusion. However, in this region, CCAO injury increased CAT activity (242.02 ± 15.77%, *^ΦΦ^P* = 0.0064) compared with the healthy and MWM groups, and the subacute prophylactic zinc administration reverted this increase (77.28 ± 6.58%, ^††^*P* = 0.0036) (Figure 4(g)). In the hippocampus, the CAT activity was not statistically different among all experimental groups (Figure 4(h)).

**Figure 5:**
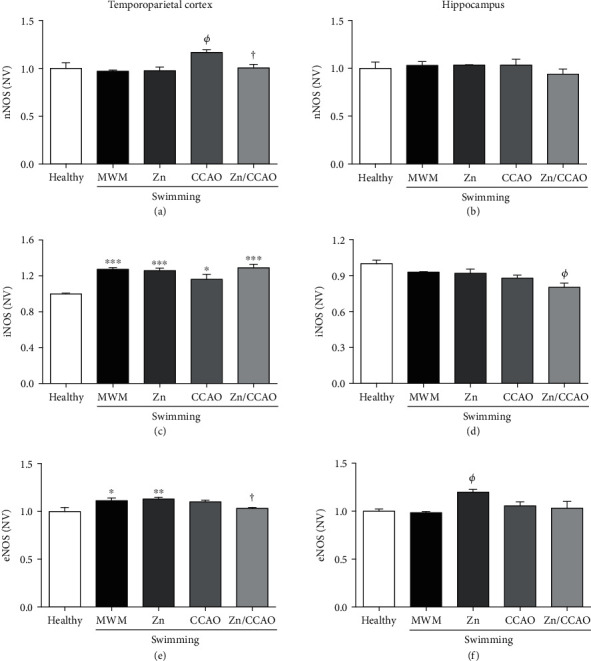
The subacute prophylactic zinc and swimming administration prevented increased nNOS and iNOS levels only in the temporoparietal cortex of rats with 30 min CCAO on day 12 postreperfusion. The values are mean ± SEM (*n* = 4) and analyzed with one-way ANOVA and Dunnett's post hoc multiple comparisons. *P* < 0.05, ^∗^compared with the healthy group, *^Φ^*compared with the MWM group and ^†^compared with the CCAO group. NV: normalized values; nNOS: neuronal nitric oxide synthase; iNOS: inducible nitric oxide synthase; eNOS: endothelial nitric oxide synthase.

ELISA measurements in the temporoparietal cortex showed significantly increased NOS levels compared with the healthy group on day 12 postreperfusion as follows, nNOS in the CCAO group (Figure 5(a)), iNOS (Figure 5(c)), and eNOS in all groups (Figure 5(e)). The subacute prophylactic zinc administration decreased the levels of nNOS (13.84 ± 3.06%, ^†^*P* = 0.0455) (Figure 5(a)) and eNOS (6.12 ± 0.92%, ^†^*P* = 0.0161) compared with the CCAO group (Figure 5(e)). In the hippocampus, no changes related to CCAO and zinc treatment were found in NOS levels on day 12 postreperfusion (Figures 5(b), 5(d), and 5(f)). However, iNOS levels were low (13.52 ± 3.61%, *^Φ^P* = 0.0316) in the Zn+CCAO group compared with the rest of the groups (Figure 5(d)), whereas increased eNOS levels (21.77 ± 2.96%, *^Φ^P* = 0.0305) were found in the Zn group (Figure 5(f)).

**Figure 6:**
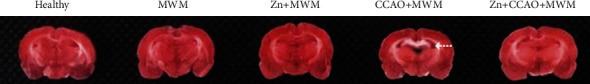
The subacute prophylactic zinc administration and swimming prevented tissue damage induced by 30 min CCAO in the hippocampus, as revealed by TTC staining. Slices of 2 mm thickness from fresh brains were stained with 2,3,5-triphenyltetrazolium chloride (TTC). Images representative of 3 brains per group. The white arrows indicate the infarct zones in the hippocampus. The arrow indicates the infarct core.

Preliminary results showed that the prophylactic zinc administration in the absence of MWM (Zn+CCAO group) caused greater damage revealed by TTC staining at 24 h and 7 days postreperfusion (data not shown). In contrast, the TTC staining in fresh brains on day 12 postreperfusion showed that the evident infarct core in the CCAO hippocampus was prevented by the prophylactic zinc administration combined with swimming (Zn+CCAO+MWM group) (Figure 6).

**Figure 7:**
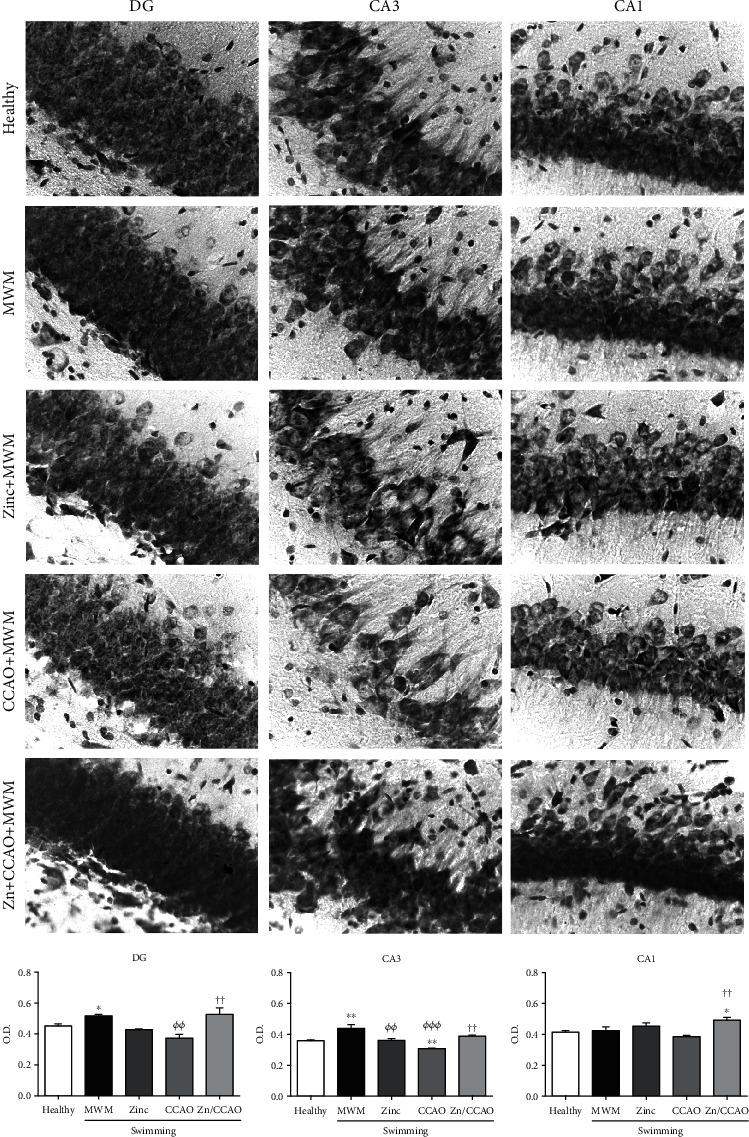
The subacute prophylactic zinc administration and swimming (Morris Water Maze, MWM) prevented tissue damage induced by 30 min CCAO in the hippocampus, as revealed by Nissl staining. O.D.: optical density. The values are mean ± SEM (*n* = 4) and analyzed with one-way ANOVA and Dunnett's post hoc multiple comparisons. *P* < 0.05, ^∗^compared with the healthy group, *^Φ^*compared with the MWM group, and ^†^compared with the CCAO group.

Confirming the tissue injury shown by TTC staining (Figure 6), Nissl staining brought about decreased optical density in the three hippocampal layers on day 12 after 30 min CCAO compared with the healthy and MWM groups (Figure 7). Meanwhile, the optical density in the three layers was normalized in the Zn+CCAO+MWM group (Figure 7). In addition, healthy rats submitted to the MWM showed a significant increase in optical density in the hippocampus CA3 layer (Figure 7).

**Figure 8:**
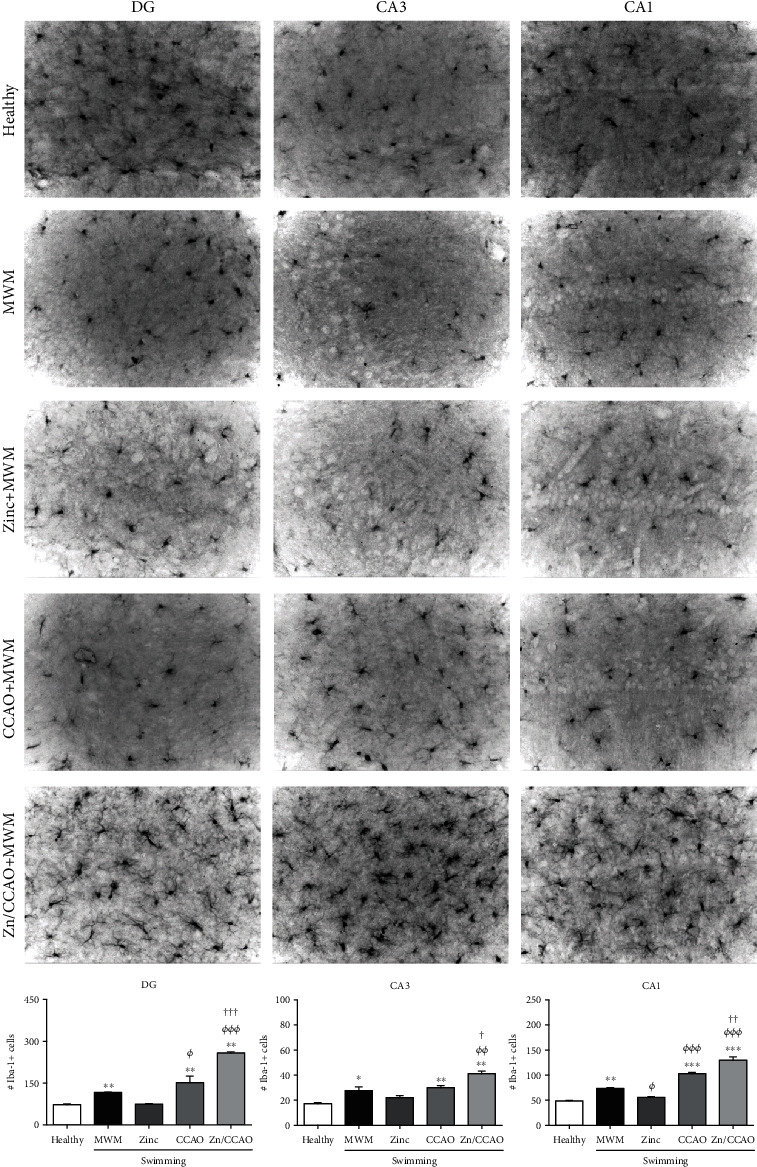
The subacute prophylactic zinc administration and swimming (Morris Water Maze, MWM) increased Iba-1 in the hippocampus of rats with 30 min CCAO on day 12 postreperfusion. The values are mean ± SEM (*n* = 4) and analyzed with one-way ANOVA and Dunnett's post hoc multiple comparisons. *P* < 0.05, ^∗^compared with the control group, *^Φ^*compared with the MWM group, and ^†^compared with the CCAO group.

Iba-1 immunohistochemistry was used to explore the response of activated microglia cells in the hippocampus. An increased number of Iba-1-positive cells was consistently found in the three hippocampal layers of the CCAO group compared with the healthy group (Figure 8). Surprisingly, a more considerable increase in Iba-1-positive number was found in the three hippocampal layers than in the CCAO group (Figure 8), possibly corresponding to the M2 phenotype microglia response (Figure 8). The number of Iba-1-positive cells was variable in the other groups; whereas it increased in the MWM group, it remained unchanged in the Zn group (Figure 8).

The HSPEPS analysis of the histopathological study showed a high score (7.5) in the MWM group compared to the healthy group (5), and this score was reduced to 5 by the subacute prophylactic zinc administration (Zn+MWM group). On the other hand, the lesser score (4) was seen in the CCAO+MWM group, revealing the ischemic damage in the hippocampal CA3 layer. Compared with the former group, the score in the Zn+CCAO+MWM group was higher (7), reaching the score in the MWM group (Table 4).

**Figure 9:**
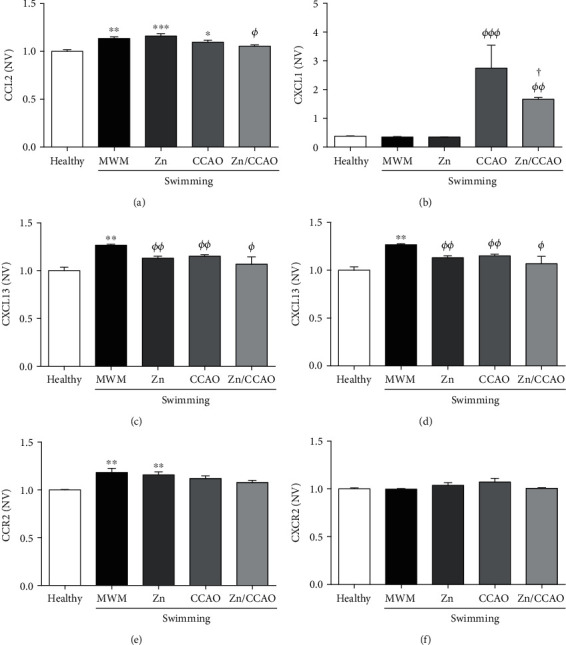
The subacute prophylactic zinc administration and swimming partially prevented the CXCL1 increase induced by 30 min CCAO in the temporoparietal cortex on day 12 postreperfusion. The values are mean ± SEM (*n* = 4) and analyzed with one-way ANOVA and Dunnett's post hoc multiple comparisons. ^∗^*P* < 0.05, compared with the control group, *^Φ^*compared with the MWM group, and ^†^compared with the CCAO group. NV: normalized values.

**Figure 10:**
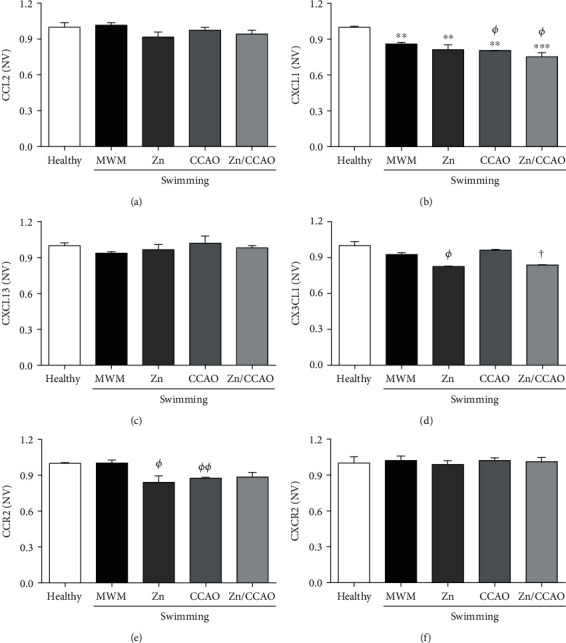
CXCL1, CX3CL1, and CCR2 levels were decreased by treatments compared with the MWM group in the hippocampus on day 12 postreperfusion The values are mean ± SEM (*n* = 4) and analyzed with one-way ANOVA and Dunnett's post hoc multiple comparisons. *P* < 0.05, ^∗^compared with the healthy group, *^Φ^*compared with the MWM group, and ^†^compared with the CCAO group. NV: normalized values.

ELISA assays showed variable chemokine levels in all experimental groups of the temporoparietal cortex (Figure 9) and hippocampus (Figure 10). Of relevance to the effect of the combined treatment on ischemia-induced changes, only CXCL1 was found significantly increased (688.14 ± 228.01%, *^ΦΦΦ^P* = 0.0001) in the temporoparietal cortex of the CCAO group compared with the healthy group (Figure 9(b)). Such an increase was prevented by the subacute prophylactic zinc treatment combined with swimming exercise but could not normalize CXCL1 levels to healthy values (Figure 9(b)).

ELISA assays showed that 30 min CCAO reduced CXCL1 by 14.08 ± 1.45% (^∗∗^*P* = 0.0095) (Figure 10(b)) and CCR2 by 14.08 ± 1.45% (^∗∗^*P* = 0.0095) (Figure 10(e)) in the hippocampus compared with the healthy group on day 12 postreperfusion. However, this decrease was unchanged by subacute prophylactic zinc administration combined with swimming (Figures 10(b) and 10(e)).

HSPEPS in biochemical studies included nitrosative stress, NOS isoforms, antioxidant activity, and chemokines. This analysis showed that swimming (MWM group) yielded a higher score (17) than the healthy group (13) that was unmodified by the subacute prophylactic zinc administration (Zn+MWM) and the cerebral ischemia (CCAO+MWM) groups. However, the subacute prophylactic zinc administration combined with a 5-day swimming exercise produced a score (13) similar to the healthy value (Table 5).

## 4. Discussion

This work focused on a transient moderate cerebral ischemia whose functional deficiency could still be at least partially reversed by treatments. In particular, the work explored the effect of subacute prophylactic zinc administration combined with swimming exercise on biochemical and cellular alterations in the late stage of ischemia. In this stage, the neurological deficit persists regardless of neuroinflammation which was naturally resolved by the end of the early stage (before two weeks postreperfusion). Our results prove that subacute prophylactic zinc administration combined with a 5-day swimming exercise in MWM avoided the cognitive and emotional deficits commonly presented in the clinic [70, 71], but not motor activity. Furthermore, preventing those deficits is consistent with reducing tissue injury and promoting neuroregeneration in the hippocampus, as TTC and Nissl staining showed. On this basis, it is thought that the increased Iba-1 cell (+) density in the hippocampus would reflect the participation of M2 phenotype microglia trying to remove cell detritus rather than the neuroinflammatory process endurance. Similarly, the increase in nitrosative stress, nNOS, iNOS, and CAT activity in the temporoparietal cortex in the CCAO group might reflect a late postischemic sequel in this region that can account for the reduced free mobility in the open field and its resistance to treatment with the subacute prophylactic zinc administration combined with swimming at 30 days postreperfusion. Preliminary studies show that 2.5 mg/kg subacute prophylactic administration in the absence of swimming caused severe cerebral damage after CCAO at 24 h and 7 days postreperfusion (data not shown). This harmful effect can be caused by the higher zinc concentration accumulated in the brain [49] due to the zinc treatment and the released zinc from presynaptic vesicles following long times of artery occlusion, thus leading to excitotoxicity [72]. On this basis, we utilized the zinc treatment combined with swimming exercise in a transient ischemic process of 30 min.

### 4.1. Swimming Exercise and Nitrosative Stress

Different modalities of swimming exercise are known to elicit a preconditioning effect [73, 74], able to attenuate or reverse cerebral injury in the early stage after ischemia [75], and improve cognition [76]. Furthermore, NO production from the three NOS isoforms [77] and increased hydroxyl radical rate [78] triggered by aerobic exercise participate in the formation of associative and spatial memory [79]. Our results in the temporoparietal cortex in the MWM group showed that swimming exercise during the late stage of ischemia increased lipid peroxidation, iNOS, and eNOS, events also associated with improved sensory and motor skills [80], spatial recognition, and long-term learning and memory [81]. Altogether, these findings support that NO plays a critical role in synaptic transmission, acting as a retrograde neurotransmitter [79], and that ROS are required in LTP formation, increasing memory acquisition and consolidation [82, 83].

We found that rats with 30 min CCAO submitted to swimming exercise presented learning and memory loss on day 12 postreperfusion, showing that swimming exercise alone was insufficient to avoid cerebral ischemic damage. In contrast, the prevention of cognitive deficits and tissue damage was achieved with the addition of subacute prophylactic zinc administration. eNOS likely mediates this beneficial effect since exercise prevents vascular endothelium dysfunction [77, 84, 85], reduces infarct volume [86], and promotes nerve repair after cerebral ischemia [77]. Furthermore, zinc stabilizing eNOS increases its enzymatic activity [87]. On the contrary, zinc deficiency increases O_2_^−^ levels by eNOS, promoting cerebral damage [88], as could occur in the CCAO group.

An increasing number of reports have associated the protective effect of zinc with swimming exercise stimulation and improved cell functioning [89, 90]. Accordingly, swimming training using MWM protects hippocampal neurons against degenerative changes and improves learning and memory [51]. Furthermore, oral supplementation of 16 or 32 ppm ZnSO_4_ [91, 92] and subacute intraperitoneal administration of 2.5 mg/kg of ZnCl_2_ [41] or 0.2 mg/kg chronically administered [42] prevent memory loss in ischemia or TBI [41, 42, 51]. The protective effect of exercise and zinc is proposed to be mediated by increasing the expression of growth factors [41] through regulating CREB [67] and antioxidant enzymes, such as SOD [42] and CAT, in animal damage models [93, 94]. These neuroprotective events are shown to occur in the early stage (day 7) postreperfusion when ROS is elevated by the ischemic process [95, 96], as confirmed by unpublished results of our group. However, in the late stage (day 12), zinc and swimming exercise did not modify SOD and CAT activity in the hippocampus but decreased the CCAO-activated CAT activity in the temporoparietal cortex. These results suggest that the increased CAT activity in the ischemic group reflects a response to oxidative stress, which is prevented by zinc combined with swimming. In addition, zinc decreases CAT activity in neonate rats [69] through the regulation of ERK phosphorylation [97].

Furthermore, zinc can exert an anxiolytic effect [98] by decreasing the inflammatory process [99], nNOS [100], and cell damage [99]; increasing antioxidant enzymes [101], BDNF [102], and synaptic plasticity; modulating neurochemical transmission [103]; regulating the activity of NMDA receptors [103–105]; and reducing the ischemic damage [106].

In addition, several studies show that the administration of NMDA receptor antagonists prevented tissue damage [107, 108] and modified some aspects of behavior [108], which can be enhanced by hypothermia caused by anesthesia [109]. In this work, we used ketamine which has been useful in models of ischemia, being capable of reducing the damage when administered superacute and therapeutically mode [110, 111], due to its inhibitor effect on glutamate receptors [112].

Accordingly, subacute prophylactic zinc administration combined with swimming increased the entry to the open arms in the elevated plus maze, thus showing that the combined treatment prevented the CCAO-induced anxiety. However, the combined treatment could not improve motor skills in the open field, as reported in the TBI model [51]. These results show that the subacute prophylactic zinc administration combined with swimming zinc partially protects the sequel of ischemic damage on day 30 after reperfusion.

### 4.2. Zinc Administration Combined with Swimming Prevents Tissue Injury

The 30 min hypoxia-ischemia model causes neuronal death that remains until seven days in the infarct core [24], whereas in the penumbra zone, highly plastic events triggered lead to tissue regeneration 14 days postreperfusion [27]. Our results agree with both events. First, the CCAO group showed signs of infarct on day 12 postreperfusion, revealed by the decreased cell density mainly in DG and CA3 layers of the hippocampus that coincides with the reduced cell viability. Second, the recovery of cell density and viability in the hippocampus in the Zn+CCAO group indicates that the subacute prophylactic zinc administration induced potentiation of plastic events. A similar effect was observed in a 10 min CCAO model [11], thus showing that the zinc neuroprotector effect combined with swimming is still effective in a cerebral injury of 30 min CCAO, reported previously in a 10 min model [11, 41, 42].

Tissue recovery from CNS injury also involves the participation of microglia [113]. The CCAO group showed an increased Iba-1(+) cell number to the healthy group on day 12 postreperfusion, suggesting that the natural course of both neuroinflammation and tissue recovery has yet not terminated on day 12 postreperfusion. However, subacute prophylactic zinc administration combined with swimming increased the Iba-1 (+) cell population over that of the CCAO group on day 12 postreperfusion. This result agrees with the previous report showing that the Iba-1 (+) cell number is still high at this time and decreased at 14 days postreperfusion [27]. However, subacute prophylactic zinc administration combined with swimming decreased the protein levels of chemokines (CXCL1, CCL2, CXCL13, and CX3CL1), the three isoforms of NOS, and the tissue damage caused by CCAO, showing a decrease in the neuroinflammatory process. These results also suggest that the combined treatment modulated the inflammation process, decreasing chemoattraction of leukocytes such as neutrophils by CXCL1 [114], macrophage/microglia by CCL2 [115] and CX3CL1 [116], and lymphocyte B, natural killer cells [117], and M1 microglia activation [118] by CXCL13 [119]. Therefore, the increase in microglial cells does not indicate a harmful effect but their participation in removing cell detritus.

In contrast, some reports have shown that these chemokines also have a neuroprotector effect in the late phase postreperfusion. For instance, CCL2 chemoattracts neuroblasts [120] and promotes neurogenesis [121] and synaptic plasticity in the hippocampus [122], thus decreasing the infarct size and recovering the cognitive function [41, 123]. In addition, CXCL1 promotes the remyelination process through the migration and maturation of oligodendrocytes [124]. Furthermore, CXCL13 induces proliferation and autophagy of mesenchymal stem cells [125] and has a chemoattractant action of neuroblast [126], and CX3CL1 causes the chemoattraction and differentiation of neural precursor cells [127] and reduces the microglia reactivity, decreasing neuroinflammation [128, 129]. However, the decrease in these chemokines by prophylactic zinc administration combined with zinc could affect those recovery processes in the long term.

The HSPEPS analysis reported previously [69] shows that in the CCAO+MWM group, the tissue damage correlates with the cognitive dysfunction, although biochemically; there are no differences with the MWM group. The MWM group showed a more beneficial effect on hippocampal cytoarchitecture and biochemical variables than the healthy control group. However, the subacute zinc prophylactic administration blocked the cognitive deficit and tissue damage caused by CCAO, and such effect was unmodified on the biochemical variables. In contrast, the score equality to the healthy control and MWM values observed in the Zn+CCAO+MWM in all the tests shows that the prophylactic zinc administration combined with swimming exercise prevented the 30 min CCAO-induced ischemic damage. In summary, HSPEPS analysis proves that the individual treatment did not cause the expected effect, i.e., a preconditioning effect by the prophylactic zinc administration or the therapeutic effect by swimming. However, effective neuroprotection against ischemic damage induced by 30 min CCAO in the rat was reached by combining the prophylactic and therapeutic strategies.

## 5. Conclusion

Prophylactic zinc administration combined with swimming exercise prevented ischemic damage in the 30 min CCAO model by decreasing neuroinflammation and nitrosative stress, preventing cell death, and improving the spatial learning-memory on day 12 postreperfusion and anxiety-like behavior on day 30 postreperfusion. These results suggest that subacute prophylactic zinc administration requires another complementary therapeutic approach to maintain the neuroprotector effect in the late phase of cerebral hypoxia-ischemia.

## Figures and Tables

**Table 1:** Performance score for each treatment per animal.

Score	Performance
0	Lower than healthy
1	Equal to healthy
1.5-2	Higher than healthy

**Table 2:** Hierarchization score of the protective effect of pharmacological strategies (HSPEPS).

Performance score	Recovering ratio
<Control	Inefficient
=Control	Efficient
>Control	Enhanced

**Table 3:** Hierarchization score of the protective effect of pharmacological strategies (HSPEPS) in cognitive test.

Cognitive test	Healthy	MWM	Zn+MWM	CCAO+MWM	Zn/CCAO+MWM
Learning	NA	1	1	0	1
Memory	NA	1	1	0	1
EPM	NA	1	1	0	1
Open field	NA	1	0	0.5	0.5
Total		4	3	0.5	3.5

NA: does not apply.

**Table 4:** Hierarchization score of the protective effect of pharmacological strategies (HSPEPS) in histopathology study.

Histopathology study	Healthy	MWM	Zn+MWM	CCAO+MWM	Zn/CCAO+MWM
TTC	1	1	1	0 hip	1
Nissl hippocampus					
DG	1	2	1	1	1
CA1	1	1	1	1	2
CA3	1	2	1	0	1
Iba-1	1	1.5	1	2	2
Total	5	7.5	5	4	7

**Table 5:** Hierarchization score of the protective effect of pharmacological strategies (HSPEPS) in biochemical studies.

Biochemical studies	Healthy	MWM	Zn+MWM	CCAO+MWM	Zn+CCAO+MWM
LPO	1	2	1.5	1.5	1.5
Nitrites	1	0	2 hip	0	0
SOD	1	1	1	1	1
CAT	1	0	0	2	0
nNOS	1	1	1	2	1
iNOS	1	2	2	2	2
eNOS	1	2	2	1	1
CCL2	1	2	2	2	1
CXCL1	1	1	1	2	1.5
CXCL13	1	2	1	1	1
CX3CL1	1	1	1	1	1
CCR2	1	2	2	1	1
CXCR2	1	1	1	1	1
Total	13	17	17.5	17.5	13

## Data Availability

Data is included in this manuscript.

## References

[1] Hu X., De Silva T. M., Chen J., Faraci F. M. (2017). Cerebral vascular disease and neurovascular injury in ischemic stroke. *Circulation Research*.

[2] WHO Stroke, Cerebrovascular accident. http://www.emro.who.int/health-topics/stroke-cerebrovascular-accident/index.html.

[3] Wardlaw J. M., Smith C., Dichgans M. (2013). Mechanisms of sporadic cerebral small vessel disease: insights from neuroimaging. *Lancet Neurology*.

[4] Xing C., Arai K., Lo E. H., Hommel M. (2012). Pathophysiologic cascades in ischemic stroke. *International Journal of Stroke*.

[5] Chavez J. C., Hurko O., Barone F. C., Feuerstein G. Z. (2009). Pharmacologic interventions for stroke: looking beyond the thrombolysis time window into the penumbra with biomarkers, not a stopwatch. *Stroke*.

[6] Shi Y., Zhang L., Pu H. (2016). Rapid endothelial cytoskeletal reorganization enables early blood-brain barrier disruption and long-term ischaemic reperfusion brain injury. *Nature Communications*.

[7] Unal-Cevik I., Gursoy-Ozdemir Y., Yemisci M. (2011). Alpha-synuclein aggregation induced by brief ischemia negatively impacts neuronal survival in vivo: a study in [A30P]alpha-synuclein transgenic mouse. *Journal of Cerebral Blood Flow and Metabolism*.

[8] Liu Y. C., Lee Y. D., Wang H. L. (2017). Anesthesia-induced hypothermia attenuates early-phase blood-brain barrier disruption but not infarct volume following cerebral ischemia. *PLoS One*.

[9] Franke M., Bieber M., Kraft P., Weber A. N. R., Stoll G., Schuhmann M. K. (2021). The NLRP3 inflammasome drives inflammation in ischemia/reperfusion injury after transient middle cerebral artery occlusion in mice. *Brain, Behavior, and Immunity*.

[10] Aguilar-Alonso P., Martinez-Fong D., Pazos-Salazar N. G. (2008). The increase in zinc levels and upregulation of zinc transporters are mediated by nitric oxide in the cerebral cortex after transient ischemia in the rat. *Brain Research*.

[11] Blanco-Alvarez V. M., Lopez-Moreno P., Soto-Rodriguez G. (2013). Subacute zinc administration and L-NAME caused an increase of NO, zinc, lipoperoxidation, and caspase-3 during a cerebral hypoxia-ischemia process in the rat. *Oxidative Medicine and Cellular Longevity*.

[12] Ginsberg M. D., Graham D. I., Busto R. (1985). Regional glucose utilization and blood flow following graded forebrain ischemia in the rat: correlation with neuropathology. *Annals of Neurology*.

[13] Tsuchiya D., Hong S., Matsumori Y. (2003). Overexpression of rat heat shock protein 70 reduces neuronal injury after transient focal ischemia, transient global ischemia, or kainic acid-induced seizures. *Neurosurgery*.

[14] Heiss W. D., Graf R., Fujita T. (1997). Early detection of irreversibly damaged ischemic tissue by flumazenil positron emission tomography in cats. *Stroke*.

[15] Welsh F. A., Ginsberg M. D., Rieder W., Budd W. W. (1978). Diffuse cerebral ischemia in the cat: II. Regional metabolites during severe ischemia and recirculation. *Annals of Neurology*.

[16] Awooda H. A., Lutfi M. F., Sharara G. G., Saeed A. M. (2015). Oxidative/nitrosative stress in rats subjected to focal cerebral ischemia/reperfusion. *Int J Health Sci (Qassim)*.

[17] Liu F., McCullough L. D. (2014). The middle cerebral artery occlusion model of transient focal cerebral ischemia. *Methods in Molecular Biology*.

[18] Sairanen T. R., Lindsberg P. J., Brenner M., Siren A. L. (1997). Global forebrain ischemia results in differential cellular expression of interleukin-1beta (IL-1beta) and its receptor at mRNA and protein level. *Journal of Cerebral Blood Flow and Metabolism*.

[19] Pawluk H., Wozniak A., Grzesk G. (2020). The role of selected pro-inflammatory cytokines in pathogenesis of ischemic stroke. *Clinical Interventions in Aging*.

[20] Pang L., Ye W., Che X. M., Roessler B. J., Betz A. L., Yang G. Y. (2001). Reduction of inflammatory response in the mouse brain with adenoviral-mediated transforming growth factor-ss1 expression. *Stroke*.

[21] Supanc V., Biloglav Z., Kes V. B., Demarin V. (2011). Role of cell adhesion molecules in acute ischemic stroke. *Annals of Saudi Medicine*.

[22] Zhou W., Liesz A., Bauer H. (2013). Postischemic brain infiltration of leukocyte subpopulations differs among murine permanent and transient focal cerebral ischemia models. *Brain Pathology*.

[23] Park J. H., Cho J. H., Ahn J. H. (2018). Neuronal loss and gliosis in the rat striatum subjected to 15 and 30 minutes of middle cerebral artery occlusion. *Metabolic Brain Disease*.

[24] Buscemi L., Price M., Bezzi P., Hirt L. (2019). Spatio-temporal overview of neuroinflammation in an experimental mouse stroke model. *Scientific Reports*.

[25] Choi B. Y., Won S. J., Kim J. H. (2018). EAAC1 gene deletion reduces adult hippocampal neurogenesis after transient cerebral ischemia. *Scientific Reports*.

[26] Dief A. E., Hassan P. S., Hartmut O., Jirikowski G. F. (2018). Neuronal and glial regeneration after focal cerebral ischemia in rat, an immunohistochemical and electron microscopical study. *Alexandria Journal of Medicine*.

[27] Li H., Zhang N., Lin H. Y. (2014). Histological, cellular and behavioral assessments of stroke outcomes after photothrombosis-induced ischemia in adult mice. *BMC Neuroscience*.

[28] Wakayama K., Shimamura M., Sata M. (2007). Quantitative measurement of neurological deficit after mild (30 min) transient middle cerebral artery occlusion in rats. *Brain Research*.

[29] Freret T., Chazalviel L., Roussel S., Bernaudin M., Schumann-Bard P., Boulouard M. (2006). Long-term functional outcome following transient middle cerebral artery occlusion in the rat: correlation between brain damage and behavioral impairment. *Behavioral Neuroscience*.

[30] Sicard K. M., Henninger N., Fisher M., Duong T. Q., Ferris C. F. (2006). Long-term changes of functional MRI-based brain function, behavioral status, and histopathology after transient focal cerebral ischemia in rats. *Stroke*.

[31] Zhong W., Yuan Y., Gu X. (2020). Neuropsychological deficits chronically developed after focal ischemic stroke and beneficial effects of pharmacological hypothermia in the mouse. *Aging and Disease*.

[32] Umarova R. M., Sperber C., Kaller C. P. (2019). Cognitive reserve impacts on disability and cognitive deficits in acute stroke. *Journal of Neurology*.

[33] Hirsch M. A., Farley B. G. (2009). Exercise and neuroplasticity in persons living with Parkinson's disease. *European Journal of Physical and Rehabilitation Medicine*.

[34] Pamplona-Santos D., Lamarao-Vieira K., Nascimento P. C. (2019). Aerobic physical exercise as a neuroprotector strategy for ethanol binge-drinking effects in the hippocampus and systemic redox status in rats. *Oxidative Medicine and Cellular Longevity*.

[35] Wu C., Yang L., Tucker D. (2018). Beneficial effects of exercise pretreatment in a sporadic Alzheimer's rat model. *Medicine and Science in Sports and Exercise*.

[36] Chali F., Desseille C., Houdebine L. (2016). Long-term exercise-specific neuroprotection in spinal muscular atrophy-like mice. *The Journal of Physiology*.

[37] Marais L., Stein D. J., Daniels W. M. (2009). Exercise increases BDNF levels in the striatum and decreases depressive-like behavior in chronically stressed rats. *Metabolic Brain Disease*.

[38] Just-Borras L., Hurtado E., Cilleros-Mane V. (2020). Running and swimming prevent the deregulation of the BDNF/TrkB neurotrophic signalling at the neuromuscular junction in mice with amyotrophic lateral sclerosis. *Cellular and Molecular Life Sciences*.

[39] Camera D. M., Smiles W. J., Hawley J. A. (2016). Exercise-induced skeletal muscle signaling pathways and human athletic performance. *Free Radical Biology & Medicine*.

[40] Kwon I., Song W., Jang Y., Choi M. D., Vinci D. M., Lee Y. (2020). Elevation of hepatic autophagy and antioxidative capacity by endurance exercise is associated with suppression of apoptosis in mice. *Annals of Hepatology*.

[41] Blanco-Alvarez V. M., Soto-Rodriguez G., Gonzalez-Barrios J. A. (2015). Prophylactic subacute administration of zinc increases CCL2, CCR2, FGF2, and IGF-1 expression and prevents the long-term memory loss in a rat model of cerebral hypoxia-ischemia. *Neural Plasticity*.

[42] Tomas-Sanchez C., Blanco-Alvarez V. M., Martinez-Fong D. (2018). Prophylactic zinc and therapeutic selenium administration increases the antioxidant enzyme activity in the rat temporoparietal cortex and improves memory after a transient hypoxia-ischemia. *Oxidative Medicine and Cellular Longevity*.

[43] Aquilani R., Baiardi P., Scocchi M. (2009). Normalization of zinc intake enhances neurological retrieval of patients suffering from ischemic strokes. *Nutritional Neuroscience*.

[44] Matsushita K., Kitagawa K., Matsuyama T. (1996). Effect of systemic zinc administration on delayed neuronal death in the gerbil hippocampus. *Brain Research*.

[45] Zhao Y. J., Yang G. Y., Domino E. F. (1996). Zinc protoporphyrin, zinc ion, and protoporphyrin reduce focal cerebral ischemia. *Stroke*.

[46] Helal G. K. (2008). Systemic administration of Zn2+ during the reperfusion phase of transient cerebral ischaemia protects rat hippocampus against iron-catalysed postischaemic injury. *Clinical and Experimental Pharmacology & Physiology*.

[47] Tomas-Sanchez C., Blanco-Alvarez V. M., Gonzalez-Barrios J. A. (2016). Prophylactic chronic zinc administration increases neuroinflammation in a hypoxia-ischemia model. *Journal of Immunology Research*.

[48] Sun B. L., An W., Xia Z. L. (2006). Zinc protoporphyrin aggravates cerebral ischemic injury following experimental subarachnoid hemorrhage. *Clinical Hemorheology and Microcirculation*.

[49] Koh J. Y., Suh S. W., Gwag B. J., He Y. Y., Hsu C. Y., Choi D. W. (1996). The role of zinc in selective neuronal death after transient global cerebral ischemia. *Science*.

[50] Morris D. R., Levenson C. W. (2013). Zinc in traumatic brain injury. *Current Opinion in Clinical Nutrition and Metabolic Care*.

[51] Cope E. C., Morris D. R., Scrimgeour A. G., VanLandingham J. W., Levenson C. W. (2011). Zinc supplementation provides behavioral resiliency in a rat model of traumatic brain injury. *Physiology & Behavior*.

[52] Cope E. C., Morris D. R., Levenson C. W. (2012). Improving treatments and outcomes: an emerging role for zinc in traumatic brain injury. *Nutrition Reviews*.

[53] Anderson C. T., Radford R. J., Zastrow M. L. (2015). Modulation of extrasynaptic NMDA receptors by synaptic and tonic zinc. *Proceedings of the National Academy of Sciences of the United States of America*.

[54] Jalali-Yazdi F., Chowdhury S., Yoshioka C., Gouaux E. (2018). Mechanisms for zinc and proton inhibition of the GluN1/GluN2A NMDA receptor. *Cell*.

[55] Jarosz M., Olbert M., Wyszogrodzka G., Mlyniec K., Librowski T. (2017). Antioxidant and anti-inflammatory effects of zinc. Zinc-dependent NF-*κ*B signaling. *Inflammopharmacology*.

[56] Zhao Y., Tan Y., Dai J. (2011). Exacerbation of diabetes-induced testicular apoptosis by zinc deficiency is most likely associated with oxidative stress, p38 MAPK activation, and p53 activation in mice. *Toxicology Letters*.

[57] Travaglia A., La Mendola D. (2017). Zinc interactions with brain-derived neurotrophic factor and related peptide fragments. *Vitamins and Hormones*.

[58] Huang Y. Z., Pan E., Xiong Z. Q., McNamara J. O. (2008). Zinc-mediated transactivation of TrkB potentiates the hippocampal mossy fiber-CA3 pyramid synapse. *Neuron*.

[59] Galvez-Peralta M., Wang Z., Bao S., Knoell D. L., Nebert D. W. (2014). Tissue-specific induction of mouse ZIP8 and ZIP14 divalent cation/bicarbonate symporters by, and cytokine response to, inflammatory signals. *International Journal of Toxicology*.

[60] Zhan J., Qin W., Zhang Y. (2016). Upregulation of neuronal zinc finger protein A20 expression is required for electroacupuncture to attenuate the cerebral inflammatory injury mediated by the nuclear factor-kB signaling pathway in cerebral ischemia/reperfusion rats. *Journal of Neuroinflammation*.

[61] von Bulow V., Dubben S., Engelhardt G. (2007). Zinc-dependent suppression of TNF-alpha production is mediated by protein kinase A-induced inhibition of Raf-1, I kappa B kinase beta, and NF-kappa B. *Journal of Immunology*.

[62] Cope E. C., Morris D. R., Gower-Winter S. D., Brownstein N. C., Levenson C. W. (2016). Effect of zinc supplementation on neuronal precursor proliferation in the rat hippocampus after traumatic brain injury. *Experimental Neurology*.

[63] Choi B. Y., Kim I. Y., Kim J. H. (2016). Zinc plus cyclo-(His-Pro) promotes hippocampal neurogenesis in rats. *Neuroscience*.

[64] Yang M., Bao D., Shi A. (2020). Zinc promotes patient-derived induced pluripotent stem cell neural differentiation via ERK-STAT signaling. *Stem Cells and Development*.

[65] Li D., Lang W., Zhou C. (2018). Upregulation of microglial ZEB1 ameliorates brain damage after acute ischemic stroke. *Cell Reports*.

[66] Chu Y., Mouat M. F., Harris R. B., Coffield J. A., Grider A. (2003). Water maze performance and changes in serum corticosterone levels in zinc- deprived and pair-fed rats. *Physiology & Behavior*.

[67] Li X., Wang L., Zhang S., Hu X., Yang H., Xi L. (2019). Timing-dependent protection of swimming exercise against d-galactose-induced aging-like impairments in spatial learning/memory in rats. *Brain Sciences*.

[68] Park S. S., Park H. S., Kim T. W., Lee S. J. (2020). Effects of swimming exercise on social isolation-induced memory impairment and apoptosis in old rats. *J Exerc Rehabil*.

[69] Gonzalez-Vazquez A., Aguilar-Peralta A. K., Tomas-Sanchez C. (2021). Taurine increases zinc preconditioning-induced prevention of nitrosative stress, metabolic alterations, and motor deficits in young rats following intrauterine ischemia. *Oxidative Medicine and Cellular Longevity*.

[70] Liu Z., Cai Y., Zhang X., Zhu Z., He J. (2018). High serum levels of malondialdehyde and antioxidant enzymes are associated with post-stroke anxiety. *Neurological Sciences*.

[71] Schottke H., Giabbiconi C. M. (2015). Post-stroke depression and post-stroke anxiety: prevalence and predictors. *International Psychogeriatrics*.

[72] Pochwat B., Nowak G., Szewczyk B. (2015). Relationship between Zinc (Zn2+) and Glutamate Receptors in the Processes Underlying Neurodegeneration. *Neural Plasticity*.

[73] Terashi T., Otsuka S., Takada S. (2019). Neuroprotective effects of different frequency preconditioning exercise on neuronal apoptosis after focal brain ischemia in rats. *Neurological Research*.

[74] Zhang Z., Li R., Zhang X. (2019). Voluntary exercise promotes neurotrophic factor and suppresses apoptosis in hippocampal ischemia. *Journal of Integrative Neuroscience*.

[75] Xing Y., Yang S. D., Dong F., Wang M. M., Feng Y. S., Zhang F. (2018). The beneficial role of early exercise training following stroke and possible mechanisms. *Life Sciences*.

[76] Farokhi-Sisakht F., Sadigh-Eteghad S., Mohaddes G., Ebrahimi-Kalan A., Karimi P., Farhoudi M. (2020). Physical and cognitive training attenuate hippocampal ischemia-induced memory impairments in rat. *Brain Research Bulletin*.

[77] Sun L., Zhuang L. P., Wu W. F. (2019). Aerobic exercise repairs neurological function after cerebral ischaemia by regulating the nitric oxide. *Anais da Academia Brasileira de Ciências*.

[78] Chen Q., Xiao D. S. (2014). Long-term aerobic exercise increases redox-active iron through nitric oxide in rat hippocampus. *Nitric Oxide*.

[79] Massaad C. A., Klann E. (2011). Reactive oxygen species in the regulation of synaptic plasticity and memory. *Antioxidants & Redox Signaling*.

[80] Wang H. R., Li J. S., Chen J., Zhang H. (2006). Effects of zinc on activity of NOS and expression of nNOS in hippocampus of acute hypoxic mice. *Zhongguo Ying Yong Sheng Li Xue Za Zhi*.

[81] Garthe A., Kempermann G. (2013). An old test for new neurons: refining the Morris water maze to study the functional relevance of adult hippocampal neurogenesis. *Frontiers in Neuroscience*.

[82] De Pasquale R., Beckhauser T. F., Hernandes M. S., Giorgetti Britto L. R. (2014). LTP and LTD in the visual cortex require the activation of NOX2. *The Journal of Neuroscience*.

[83] Serrano F., Klann E. (2004). Reactive oxygen species and synaptic plasticity in the aging hippocampus. *Ageing Research Reviews*.

[84] Leardini-Tristao M., Borges J. P., Freitas F. (2017). The impact of early aerobic exercise on brain microvascular alterations induced by cerebral hypoperfusion. *Brain Research*.

[85] Ally A., Maher T. J. (2008). Endothelial NOS expression within the ventrolateral medulla can affect cardiovascular function during static exercise in stroked rats. *Brain Research*.

[86] Arrick D. M., Yang S., Li C., Cananzi S., Mayhan W. G. (2014). Vigorous exercise training improves reactivity of cerebral arterioles and reduces brain injury following transient focal ischemia. *Microcirculation*.

[87] Abregú F. M., Gobetto M. N., Juriol L. V. (2018). Developmental programming of vascular dysfunction by prenatal and postnatal zinc deficiency in male and female rats. *The Journal of Nutritional Biochemistry*.

[88] Santhanam A. V., d'Uscio L. V., Smith L. A., Katusic Z. S. (2012). Uncoupling of eNOS causes superoxide anion production and impairs NO signaling in the cerebral microvessels of hph-1 mice. *Journal of Neurochemistry*.

[89] Bicer M., Akil M., Sivrikaya A., Kara E., Baltaci A. K., Mogulkoc R. (2011). Effect of zinc supplementation on the distribution of various elements in the serum of diabetic rats subjected to an acute swimming exercise. *Journal of Physiology and Biochemistry*.

[90] Chen T. I., Chen M. Y. (2016). Zinc is indispensable in exercise-induced cardioprotection against intermittent hypoxia-induced left ventricular function impairment in rats. *PLoS One*.

[91] Piechal A., Blecharz-Klin K., Pyrzanowska J., Widy-Tyszkiewicz E. (2016). Influence of long-term zinc administration on spatial learning and exploratory activity in rats. *Biological Trace Element Research*.

[92] Piechal A., Blecharz-Klin K., Pyrzanowska J., Widy-Tyszkiewicz E. (2012). Maternal zinc supplementation improves spatial memory in rat pups. *Biological Trace Element Research*.

[93] Bashandy S. A. E., Alaamer A., Moussa S. A. A., Omara E. A. (2018). Role of zinc oxide nanoparticles in alleviating hepatic fibrosis and nephrotoxicity induced by thioacetamide in rats. *Canadian Journal of Physiology and Pharmacology*.

[94] Sefi M., Chaabane M., Elwej A. (2020). Zinc alleviates maneb-induced kidney injury in adult mice through modulation of oxidative stress, genotoxicity, and histopathological changes. *Environmental Science and Pollution Research International*.

[95] Candelario-Jalil E., Mhadu N. H., Al-Dalain S. M., Martinez G., Leon O. S. (2001). Time course of oxidative damage in different brain regions following transient cerebral ischemia in gerbils. *Neuroscience Research*.

[96] Matsuda S., Umeda M., Uchida H., Kato H., Araki T. (2009). Alterations of oxidative stress markers and apoptosis markers in the striatum after transient focal cerebral ischemia in rats. *Journal of Neural Transmission (Vienna)*.

[97] Patrushev N., Seidel-Rogol B., Salazar G. (2012). Angiotensin II requires zinc and downregulation of the zinc transporters ZnT3 and ZnT10 to induce senescence of vascular smooth muscle cells. *PLoS One*.

[98] Samardzic J., Savic K., Stefanovic N. (2013). Anxiolytic and antidepressant effect of zinc on rats and its impact on general behavioural parameters. *Vojnosanitetski Pregled*.

[99] Cavalcanti C. L., Goncalves M. C. R., Alves A. F. (2020). Antidepressant, anxiolytic and neuroprotective activities of two zinc compounds in diabetic rats. *Frontiers in Neuroscience*.

[100] Wang H. R., Li J. S., Chen J., Zhang H. (2006). Effects of taurine and zinc on activity of NOS and expression of nNOS in cerebral cortex of acute hypoxic mice. *Wei Sheng Yan Jiu*.

[101] Famitafreshi H., Karimian M. (2019). Modulation of catalase, copper and zinc in the hippocampus and the prefrontal cortex in social isolation-induced depression in male rats. *Acta Neurobiologiae Experimentalis (Wars)*.

[102] Mlyniec K., Starowicz G., Gawel M., Frackiewicz E., Nowak G. (2016). Potential antidepressant-like properties of the TC G-1008, a GPR39 (zinc receptor) agonist. *Journal of Affective Disorders*.

[103] Doboszewska U., Wlaz P., Nowak G., Radziwon-Zaleska M., Cui R., Mlyniec K. (2017). Zinc in the monoaminergic theory of depression: its relationship to neural plasticity. *Neural Plasticity*.

[104] Zhu J., Shao C. Y., Yang W. (2012). Chronic zinc exposure decreases the surface expression of NR2A-containing NMDA receptors in cultured hippocampal neurons. *PLoS One*.

[105] Szewczyk B., Poleszak E., Wlaz P. (2009). The involvement of serotonergic system in the antidepressant effect of zinc in the forced swim test. *Progress in Neuro-Psychopharmacology & Biological Psychiatry*.

[106] Sorensen J. C., Mattsson B., Andreasen A., Johansson B. B. (1998). Rapid disappearance of zinc positive terminals in focal brain ischemia. *Brain Research*.

[107] Yu G., Wu F., Wang E. S. (2015). BQ-869, a novel NMDA receptor antagonist, protects against excitotoxicity and attenuates cerebral ischemic injury in stroke. *International Journal of Clinical and Experimental Pathology*.

[108] Gerriets T., Stolz E., Walberer M., Kaps M., Bachmann G., Fisher M. (2003). Neuroprotective effects of MK-801 in different rat stroke models for permanent middle cerebral artery occlusion: adverse effects of hypothalamic damage and strategies for its avoidance. *Stroke*.

[109] Liu C. W., Liao K. H., Tseng H., Wu C. M., Chen H. Y., Lai T. W. (2020). Hypothermia but not NMDA receptor antagonism protects against stroke induced by distal middle cerebral arterial occlusion in mice. *PLoS One*.

[110] Xiong Z., Chang L., Qu Y. (2020). Neuronal brain injury after cerebral ischemic stroke is ameliorated after subsequent administration of R-ketamine, but not S-ketamine. *Pharmacology, Biochemistry, and Behavior*.

[111] Abdoulaye I. A., Wu S. S., Chibaatar E. (2021). Ketamine induces lasting antidepressant effects by modulating the NMDAR/CaMKII-mediated synaptic plasticity of the hippocampal dentate gyrus in depressive stroke model. *Neural Plasticity*.

[112] Aleksandrova L. R., Phillips A. G., Wang Y. T. (2017). Antidepressant effects of ketamine and the roles of AMPA glutamate receptors and other mechanisms beyond NMDA receptor antagonism. *Journal of Psychiatry & Neuroscience*.

[113] Li X., Chen S., Mao L. (2019). Zinc improves functional recovery by regulating the secretion of granulocyte colony stimulating factor from microglia/macrophages after spinal cord injury. *Frontiers in Molecular Neuroscience*.

[114] Losy J., Zaremba J., Skrobanski P. (2005). CXCL1 (GRO-alpha) chemokine in acute ischaemic stroke patients. *Folia Neuropathologica*.

[115] Roberts T. K., Eugenin E. A., Lopez L. (2012). CCL2 disrupts the adherens junction: implications for neuroinflammation. *Laboratory Investigation*.

[116] Lauro C., Chece G., Monaco L. (2019). Fractalkine modulates microglia metabolism in brain ischemia. *Frontiers in Cellular Neuroscience*.

[117] Gan Y., Liu Q., Wu W. (2014). Ischemic neurons recruit natural killer cells that accelerate brain infarction. *Proceedings of the National Academy of Sciences of the United States of America*.

[118] Pabon M. M., Bachstetter A. D., Hudson C. E., Gemma C., Bickford P. C. (2011). CX3CL1 reduces neurotoxicity and microglial activation in a rat model of Parkinson's disease. *Journal of Neuroinflammation*.

[119] Chen F., Li X., Li Z., Zhou Y., Qiang Z., Ma H. (2020). The roles of chemokine (C-X-C motif) ligand 13 in spinal cord ischemia-reperfusion injury in rats. *Brain Research*.

[120] Yan Y. P., Sailor K. A., Lang B. T., Park S. W., Vemuganti R., Dempsey R. J. (2007). Monocyte chemoattractant protein-1 plays a critical role in neuroblast migration after focal cerebral ischemia. *Journal of Cerebral Blood Flow and Metabolism*.

[121] Andres R. H., Choi R., Pendharkar A. V. (2011). The CCR2/CCL2 interaction mediates the transendothelial recruitment of intravascularly delivered neural stem cells to the ischemic brain. *Stroke*.

[122] Bray J. G., Reyes K. C., Roberts A. J., Ransohoff R. M., Gruol D. L. (2013). Synaptic plasticity in the hippocampus shows resistance to acute ethanol exposure in transgenic mice with astrocyte-targeted enhanced CCL2 expression. *Neuropharmacology*.

[123] Stowe A. M., Wacker B. K., Cravens P. D. (2012). CCL2 upregulation triggers hypoxic preconditioning-induced protection from stroke. *Journal of Neuroinflammation*.

[124] Vora P., Pillai P., Mustapha J. (2012). CXCL1 regulation of oligodendrocyte progenitor cell migration is independent of calcium signaling. *Experimental Neurology*.

[125] Torrano J., Al Emran A., Hammerlindl H., Schaider H. (2019). Emerging roles of H3K9me3, SETDB1 and SETDB2 in therapy-induced cellular reprogramming. *Epigenetics*.

[126] Chapman K. Z., Ge R., Monni E. (2015). Inflammation without neuronal death triggers striatal neurogenesis comparable to stroke. *Neurobiology of Disease*.

[127] Turbic A., Leong S. Y., Turnley A. M. (2011). Chemokines and inflammatory mediators interact to regulate adult murine neural precursor cell proliferation, survival and differentiation. *PLoS One*.

[128] Hulshof S., van Haastert E. S., Kuipers H. F. (2003). CX3CL1 and CX3CR1 expression in human brain tissue: noninflammatory control versus multiple sclerosis. *Journal of Neuropathology and Experimental Neurology*.

[129] Chen X., Jiang M., Li H. (2020). CX3CL1/CX3CR1 axis attenuates early brain injury via promoting the delivery of exosomal microRNA-124 from neuron to microglia after subarachnoid hemorrhage. *Journal of Neuroinflammation*.

